# Supplementary material for: Local conditions and policy design determine whether ecological compensation can achieve No Net Loss goals
Source: Nat Commun. 2020 Apr 29;11:2072. doi: 10.1038/s41467-020-15861-1 (PMC7190705; doi:10.1038/s41467-020-15861-1)
Supplement: Supplementary file 1 — Supplementary Information [file 41467_2020_15861_MOESM1_ESM.pdf]

SUPPLEMENTARY MATERIALS

Sonter et al.

Local conditions and policy design determine whether ecological compensation can achieve No Net Loss goals

FILE CONTAINS:

- Supplementary Figures 1–8
- Supplementary Methods 1–4
- Supplementary Tables 1–25
- References cited in Supplementary Materials

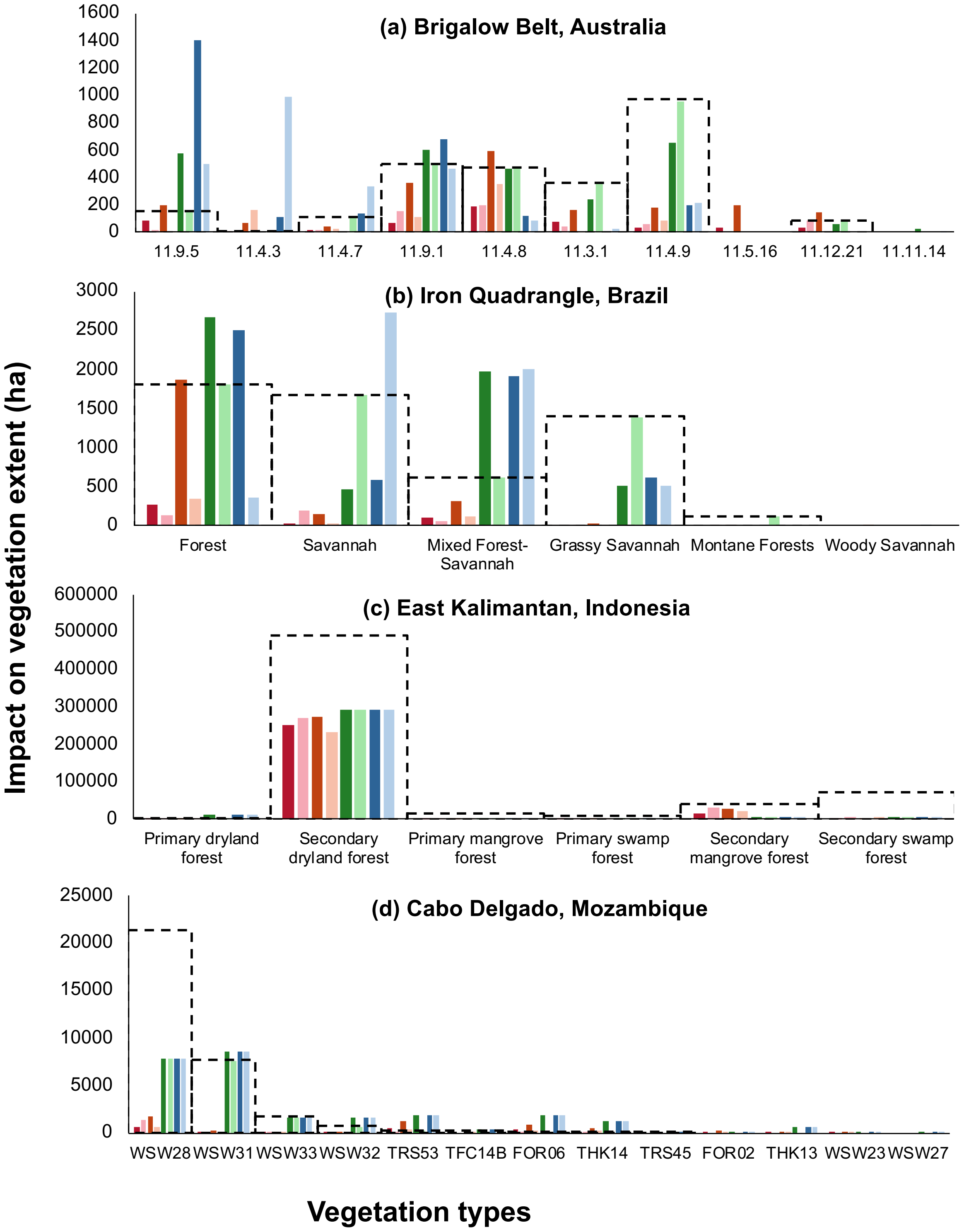

Supplementary Figure 1: Impacts of regulated development (dashed columns) and compensation policy design options (coloured bars) on native vegetation by types (see Supplementary Information A-D for descriptions of vegetation types). Policy design options described in Figure 2. Graphs show results when prioritising compensation activities Outside PAs. Panels represent four case studies: (a) Brigalow Belt, Australia; (b) Iron Quadrangle, Brazil; (c) East Kalimantan, Indonesia; (d) Cabo Delgado, Mozambique.

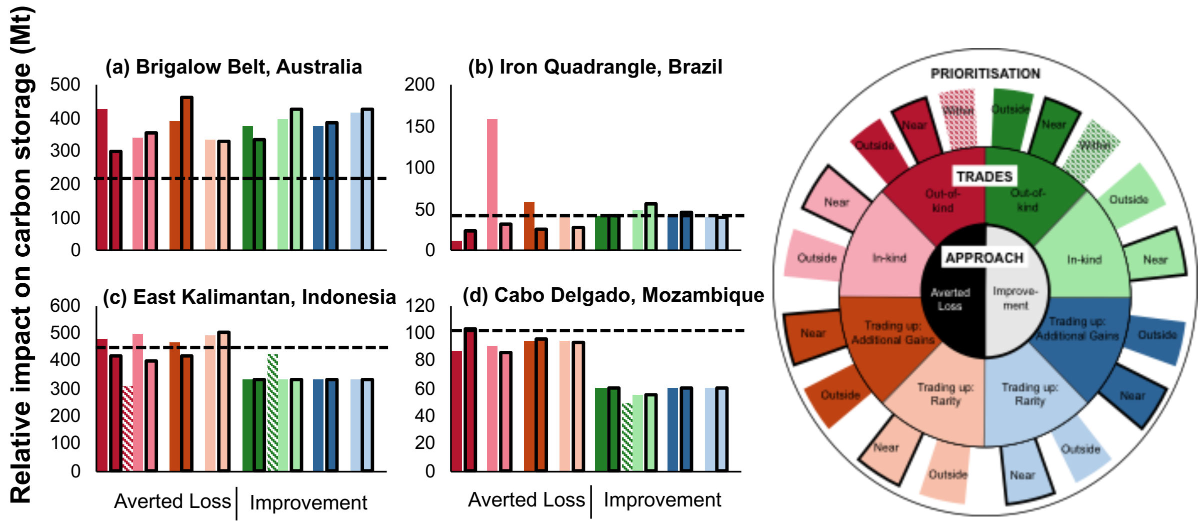

Supplementary Figure 2: Impacts of regulated development (dashed black line) and 18 policy design options (coloured bars) on carbon storage (Mt C) per impact on biodiversity (native vegetation extent; Figure 2). Policy design options described in Figure 2. Panels represent four case studies: (a) Brigalow Belt, Australia; (b) Iron Quadrangle, Brazil; (c) East Kalimantan, Indonesia; (d) Cabo Delgado, Mozambique.

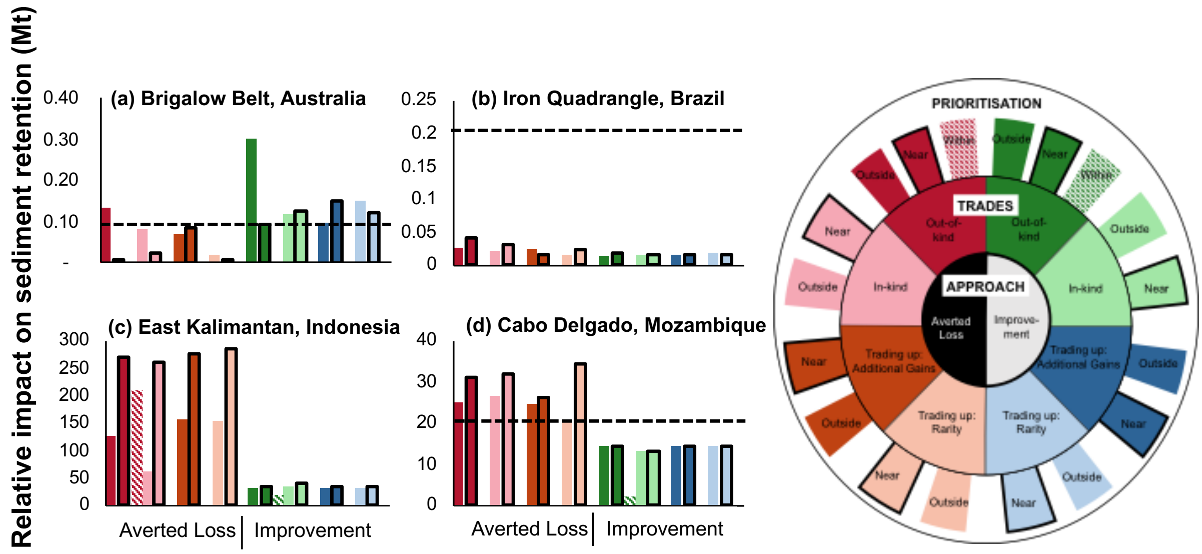

Supplementary Figure 3: Impacts of regulated development (dashed black line) and 18 policy design options (coloured bars) on sediment retention (Mt) per impact on biodiversity (native vegetation extent; Figure 2). Policy design options described in Figure 2. Panels represent four case studies: (a) Brigalow Belt, Australia; (b) Iron Quadrangle, Brazil; (c) East Kalimantan, Indonesia; (d) Cabo Delgado, Mozambique.

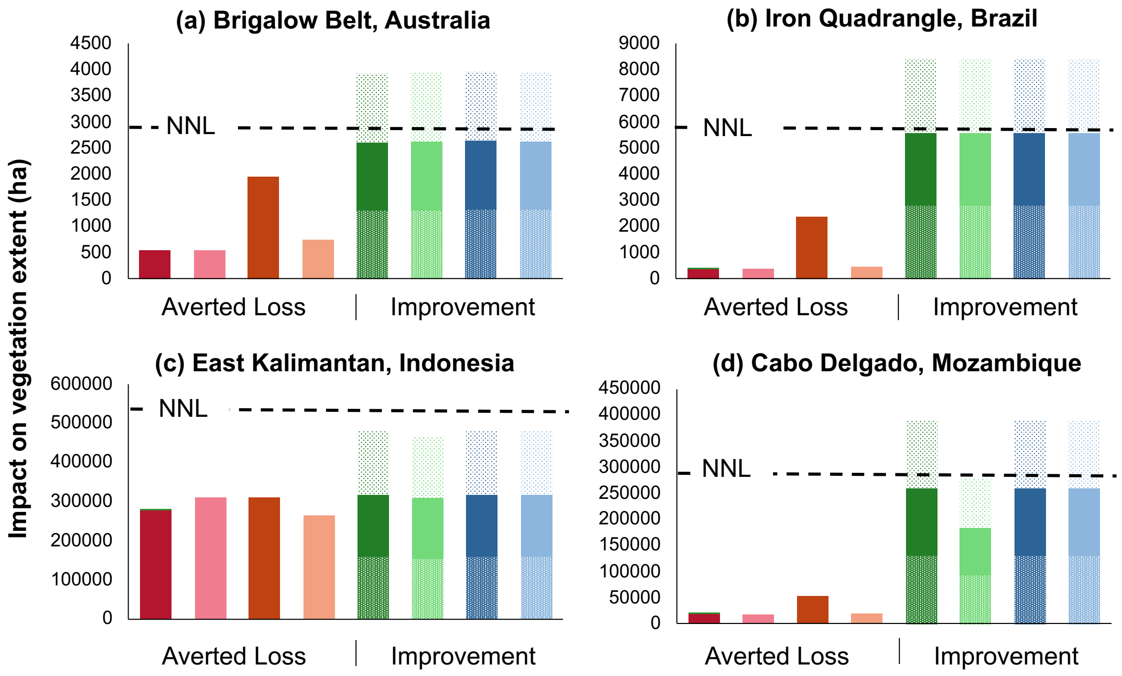

Supplementary Figure 4: Impacts of regulated development (dashed black line) and policy design options (coloured bars – when allocating compensation activities outside current PAs) on biodiversity. . Policy design options described in Figure 2. For Improvement approaches, stacked bars represent cumulative impacts under three alternative restoration success assumptions: 25% success rate, 50% success rate (as shown in the main text), 75% success rate. Panels represent four case studies: (a) Brigalow Belt, Australia; (b) Iron Quadrangle, Brazil; (c) East Kalimantan, Indonesia; (d) Cabo Delgado, Mozambique.

Supplementary Methods 1: Brigalow Belt, Australia

The Australian case study is defined by the northern extent of pre-clearing Brigalow woodlands (~2.6 Mha; Supplementary Figure 5; Supplementary Table 1). The Brigalow Belt intersects one of Australia's most productive coal mining regions and has been heavily cleared over the past century for cattle grazing[1]. While less than 42% of remnant Brigalow woodlands remain considerable regrowth occurs each year, where regrowth resembles remnant communities in term of species richness and vegetation structure after 30 years[2, 3]. Remnant Brigalow is protected under state and federal legislation[4, 5]; however, clearing for extractive projects is permitted. Recently approved mining projects fall within the Abbot Point and Galilee Basin State Development Areas[6]. Remnant vegetation cleared by these projects require some form of compensation[7, 8] and thus represent regulated development in this case study. We assumed all current vegetation within the State Development Areas[6] would be cleared.

We obtained 100 m resolution land cover maps from years 2006, 2009 and 2011[9] and reclassified them to depict current (2011) remnant vegetation, woody regrowth and cleared land, using a 12% foliage projective cover (FPC) threshold to distinguish cleared land from woody regrowth. The counterfactual land use change model was calibrated to simulate four transitions (Supplementary Table 2) according to nine spatial determinants (Supplementary Table 3). We validated the model by simulating transitions between 2009 and 2011 and comparing simulations with observed land use in 2011 and a null model. The calibrated model performed better than a null model across all transitions (Supplementary Table 4)[10]. We simulated land use transitions to the year 2020, assuming transition rates equalled those observed between years 2006 and 2009 (Supplementary Table 2), to determine the extent and distribution of counterfactual vegetation clearing and regrowth. This timeframe was used, rather than the more recent 2009–2011 period, because regrowth and clearing rates of Brigalow were unusually high during this timeframe[11] and so we chose to use the more conservative rate. However, using higher rates of clearing would result in greater performance of Averted Loss approaches[10]. To quantify impacts of development and compensation, we used pre-clearing Regional Ecosystems[12] to disaggregate current (2011) and future (2020) land cover maps by vegetation type (Supplementary Table 1).

To assess impacts on above ground carbon storage, we used the VAST model, which calculates pre-clearing carbon density (t/ha) in above and below ground carbon pools[13, 14]. We used above ground carbon pools (sum of leaf, wood, fine litter, course litter) and assumed cleared areas would have no above ground carbon. We used the following datasets to calibrate the sediment retention model: DEM[15], rainfall erosivity and soil erodibility[16], land use and land cover[17], watersheds[18], cover management and supporting practice factors[19].

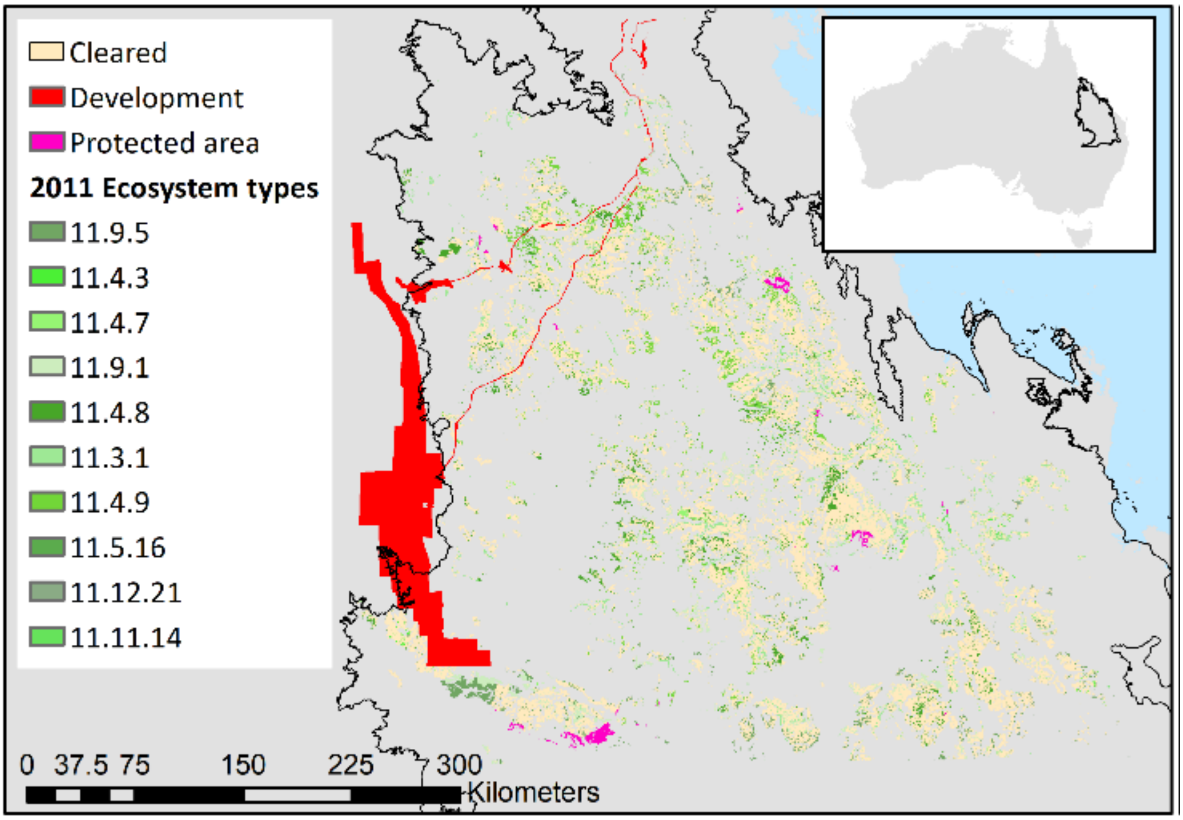

Supplementary Figure 5: Brigalow Belt, Australia [2.6 Mha]. Map shows the extent of future regulated development requiring compensation and the current extent of protected areas and native vegetation types (our indicator of biodiversity; see Supplementary Table 1). Grey areas within study region boundary denote sites that did not have historic Brigalow ecosystems and are thus excluded from our case study.

Supplementary Table 1: Regional Ecosystems (indicating vegetation types) listed as threatened Brigalow woodlands[12, 20].

**Vegetation type**	**Description**	**Extent (ha)**	
		**Historical**	**Current**
11.11.14	Acacia harpophylla open forest on deformed and metamorphosed sediments and interbedded volcanics	19646	7767
11.12.21	Acacia harpophylla open forest on igneous rocks; colluvial lower slopes	25388	8220
11.3.1	Acacia harpophylla and/or Casuarina cristata open forest on alluvial plains	452947	154614
11.4.3	Acacia harpophylla and/or Casuarina cristata shrubby open forest on Cainozoic clay plains	25513	4487
11.4.7	Open forest of Eucalyptus populnea with Acacia harpophylla or Casuarina cristata on Cainozoic clay plains	21862	5401
11.4.8	Eucalyptus cambageana open forest with Acacia harpophylla or A. argyrodendron on Cainozoic clay plains	616453	167857
11.4.9	Acacia harpophylla shrubby open forest with Terminalia oblongata on Cainozoic clay plains	664501	171856
11.5.16	Acacia harpophylla or Casuarina cristata open forest in depressions on Cainozoic sand plains or surfaces	12106	5660
11.9.1	Acacia harpophylla-Eucalyptus cambageana open forest on Cainozoic fine-grained sedimentary rocks	408534	114939
11.9.5	Acacia harpophylla and/or Casuarina cristata open forest on Cainozoic fine-grained sedimentary rocks	377119	120856

Supplementary Table 2: Land use transitions in Brigalow Belt.

From	To	Transition rates				Compensation model
		2006-2009		2009-2011		
		Total (ha)	Annual (%)	Total (ha)	Annual (%)	
Remnant	Regrowth	634	0.07	3594	0.58	No change
Remnant	Cleared	432	0.05	2529	0.42	Unregulated losses
Regrowth	Cleared	3811	0.41	6012	0.73	Unregulated losses
Cleared	Regrowth*	107444 (3811)	(0.06)	100568 (6012)	(0.16)	Gains

*Values in parenthesise indicate corrected cleared to regrowth rates when assuming the absolute area of cleared to regrowth equals that of regrowth to cleared[10]. This correction accounts for the unlikely high amount of regrowth detected by our methods, due to seasonal effects.

Supplementary Table 3: Spatial determents used to calibrate land use change model in Brigalow Belt. Variables were not auto-correlated. See[10] for more information on model calibration process.

Spatial determinants	Data source
Protected areas	[12]
Pre-clearing Regional Ecosystem classification	[12]. See Supplementary Table 1.
Soil type	[21]
Elevation	[22]
Proximity to roads	[23]
Proximity to watercourses	[23]
Proximity to cleared land	Calculated from land cover (Supplementary Table 2) and updated in the model at annual time steps.
Proximity to regrowth	
Proximity to remnant	

Supplementary Table 4: Model calibration scores for individual land use transitions in Brigalow Belt. Model performance was calculated as the accuracy of the calibrated model divided by the accuracy of the null model.

From	To	Accuracy at 100 m resolution		
		Calibration	Null	Performance
Remnant	Regrowth	0.024	0.014	1.71
Remnant	Cleared	0.008	0.007	1.14
Regrowth	Cleared	0.248	0.019	13.05
Cleared	Regrowth	0.111	0.050	2.22

Supplementary Methods 2: Iron Quadrangle, Brazil

The Iron Quadrangle covers 1.9 Mha of Minas Gerais in Brazil (Supplementary Figure 6), represents an important mining region (producing 60% of Brazil's iron ore[24]) and is located within the Atlantic Forest, one of the world's most threatened yet bio-diverse ecosystems[25]. Native vegetation has been cleared extensively for multiple purposes and clearing is expected to continue over the next 30 years, due to mine expansion[26]. These impacts will negatively affect many ecosystems closely associated with metal-rich soils and that are now of high conservation concern[27, 28]. Mines are legally required to compensate for their impacts on biodiversity[29, 30] and thus represented our regulated land use. We quantified vegetation cleared by mining using the land-use change model (described below) and overlaying these maps with maps of current native vegetation extent (Supplementary Table 5).

We obtained 100 m resolution land use maps of years 2000, 2004 and 2010[26] to determine current (2010) extent of five classes: mining, urban, plantations (often Eucalyptus monocultures, used for pulp and charcoal), cleared land (grazing or abandoned agricultural fields), and native vegetation including forests and savannas; [31]. The land use model simulated seven transitions (Supplementary Table 6), according to seven spatial determinants (Supplementary Table 7). We validated the model by simulating transitions between 2004 and 2010 and comparing simulations with observed land use in 2010 and a null model. The calibrated model performed better than a null model across all transitions (Supplementary Table 8). We simulated land use change to the year 2020, assuming transition rates equalled either historical rates (for transitions other than mine expansion) or increased linearly with projected steel production[26, 32], to determine the extent and distribution of regulated impacts and counterfactual biodiversity losses and gains. To assess impacts of development and compensation, we used historical vegetation distributions[33] to disaggregate 2010 and 2020 vegetation into native vegetation types (Supplementary Table 5).

To assess carbon storage, we used a pre-clearing map of above ground biomass (t/ha)[34] and assumed cleared areas would have no above ground biomass. We used the following datasets to model sediment retention: DEM[35]; land use and land cover[26]; rainfall erosivity, calculated using NetErosividade[36]; soil erodibility (Supplementary Table 9), cover management and supporting practice factors (Supplementary Table 10)[37]. Soil erodibility was mapped using the soil map for Minas Gerais[38].

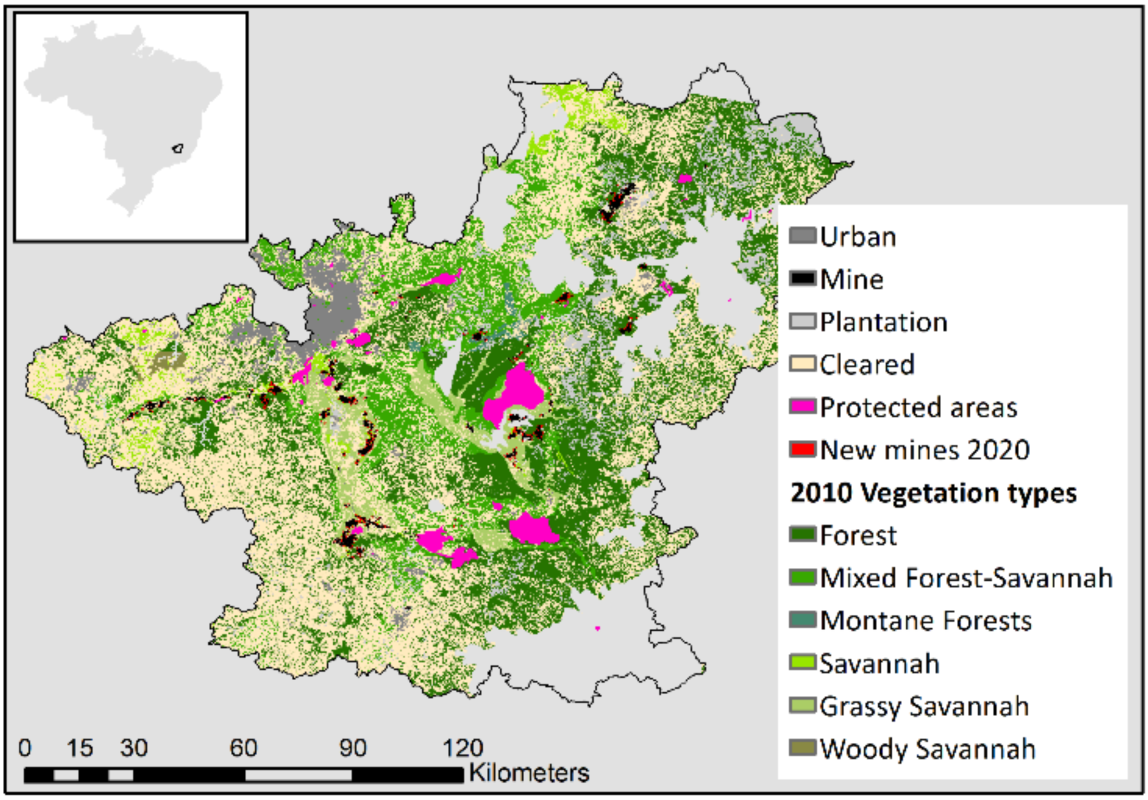

Supplementary Figure 6: Iron Quadrangle, Brazil (1.9 Mha). Map shows the extent of future regulated development requiring compensation (i.e. new mines 2020) and the current extent of protected areas and native vegetation types (our indicator of biodiversity; see Supplementary Table 5). Grey areas within study region boundary denote no data (generally due to cloud occurrence in land cover maps).

Supplementary Table 5: Native vegetation extent for historical and current (2010) conditions[31, 33] in Iron Quadrangle. Vegetation is classified according to the system originally proposed by[39].

**Vegetation types**	**Original classification**	**Extent (ha)**	
		**Historical**	**Current**
Forest	Floresta estacional semidecidual	1060894	487267
Mixed Forest-Savanna	Contato savana/floresta estacional	442923	199019
Montane Forests	Floresta estacional semidecidual montana	7280	4827
Savanna	Savana parque sem/com floresta-de-galeria	101188	30596
Grassy Savanna	Savana gramíneo-lenhosa sem/com floresta-de-galeria	106332	72241
Woody Savanna	Savana arborizada com floresta-de-galeria	4530	3943

Supplementary Table 6: Land use transitions used to calibrate and validate counterfactual model for Iron Quadrangle.

**From**	**To**	**Observed transition rates**				**Compensation model**
		**2000-2004**		**2004-2010**		
		**Total (ha)**	**Annual (%)**	**Total (ha)**	**Annual (%)**	
Cleared	Urban	1687	0.03	2988	0.06	No change
	Mining	406	0.00	550	0.01	No change
	Plantation	4961	0.11	15348	0.34	No change
	Vegetation	1115	0.02	1513	0.03	Gains
Vegetation	Cleared	8319	0.16	13881	0.28	Unregulated losses
	Urban	340	0.00	876	0.01	Unregulated losses
	Mining	1390	0.02	3384	0.07	Regulated losses

Supplementary Table 7: Spatial determinants used to calibrate land-use change model for Iron Quadrangle. Variables were not auto correlated. See [26] for more information on model calibration process.

Spatial determinants	Data source
Protected areas	[40]
Slope	[35]
Presence of mining leases	[41]
Proximity to cleared	Calculated from land use maps[26] and updated in the model at annual time steps.
Proximity to urban	
Proximity to mining	
Proximity to vegetation	

Supplementary Table 8: Model calibration scores for individual land use transitions for Iron Quadrangle. Model performance was calculated as the accuracy of the calibrated model divided by the accuracy of the null model.

From	To	Accuracy at 100 m resolution		
		Calibration	Null	Performance
Cleared	Urban	0.062	0.006	10.3
	Mining	0.074	0.002	37.0
	Plantation	0.031	0.026	1.19
	Vegetation	0.006	0.003	2.00
Vegetation	Cleared	0.042	0.025	1.68
	Urban	0.046	0.001	46.0
	Mining	0.071	0.001	71.0

Supplementary Table 9: Soil erodibility values for Iron Quadrangle[37]. Mean values combined from multiple sources[42, 43, 44, 45, 46, 47] and were applied to a soil map[48].

Soil type	K
Argisol	0.044
Cambisol	0.023
Red Latosol	0.009
Yellow-red Latosol	0.017
Fluvic Neosol	0.042
Litholic Neosol	0.045
Quartzipsamment Neosol	0.144

Supplementary Table 10: Crop and management (C) and supporting practices (P) factor for Iron Quadrangle[37] combined data from multiple sources[49, 50, 51].

Land use	C.P
Urban	0.1
Mining	1
Plantation	0.016
Vegetation	0.012
Cleared	0.052

Supplementary Methods 3: East Kalimantan, Indonesia

East Kalimantan is one of five provinces in Indonesian Borneo; 6.7 Mha remains forested, 33% as primary forest, and is home to endangered species such as the Bornean Orangutan (Pongo pygmaeus)[52]. These forests, however, face increasing pressures from a provincial economy heavily reliant on expanding palm oil production, timber plantations, mining and commercial logging[53, 54] and from associated large-scale infrastructure projects meant to accelerate development of these industrial sectors[52]. The emerging evidence, at least within the context of oil palm development, of the impractically high costs of repairing impaired landscapes to balance against losses from development[55] has raised the profile of sustainability standards and the role they can play in advancing sustainable development. In Indonesia, few sectors are as far along in the development of these standards as oil palm. The dominant certification systems for palm oil are the Roundtable on Sustainable Palm Oil (RSPO), established in 2004 through an international multi-stakeholder organization, and the Indonesian Sustainable Palm Oil Certification System (ISPO) launched by the Government of Indonesia in 2011[56]. Although implementation and enforcement of RSPO criteria (the more stringent of the two standards) has received criticism, in principle, oil palm from a plantation cannot be certified until forest clearance has been accounted for and adequate compensation made.

We obtained 30 m resolution land use/land cover maps to represent historical (1996, 2006) and current (2015) landscape conditions[57] and reclassified maps into eight land use classes at 100 m resolution (Supplementary Table 11). The counterfactual land-use change model was calibrated to simulate 15 transitions (Supplementary Table 12) according to 10 spatial determinants (Supplementary Table 14). We validated the model by simulating transitions between 2006 and 2015 and comparing simulations with observed land use in 2015 and a null model. The calibrated model performed better than a null model across all transitions (Supplementary Table 14). We simulated land-use change to the year 2040, assuming transition rates equal to those observed between years 2006 and 2015 (Supplementary Table 12), to determine the extent and distribution of counterfactual clearing and regrowth. To quantify impacts of development and compensation, we used historic (1996) land cover maps[57] to disaggregate current (2016) and future (2040) primary and secondary forests into six specific vegetation types (Supplementary Table 15).

For carbon storage, we used maps of above ground carbon storage of intact and degraded forests across East Kalimantan [58]. We used this data to estimate average above ground carbon storage for vegetation types in 2015 (Supplementary Table 16) and calculated impacts of development and compensation, assuming degraded land will have average carbon density of shrublands (rather than bare ground), either wet or dry depending on historic (1996) vegetation types, and restoration returns carbon density to that of a secondary forests (either wet or dry). We used the following datasets to model sediment retention: DEM[35], which was resampled to 100 m and then pit-filled in ArcGIS using 75 m threshold; rainfall erosivity and soil erodibility[59], land use and land cover, watersheds (delineated from DEM, using minimum drainage area of 200 km), cover management and supporting practice factors (Supplementary Table 17).

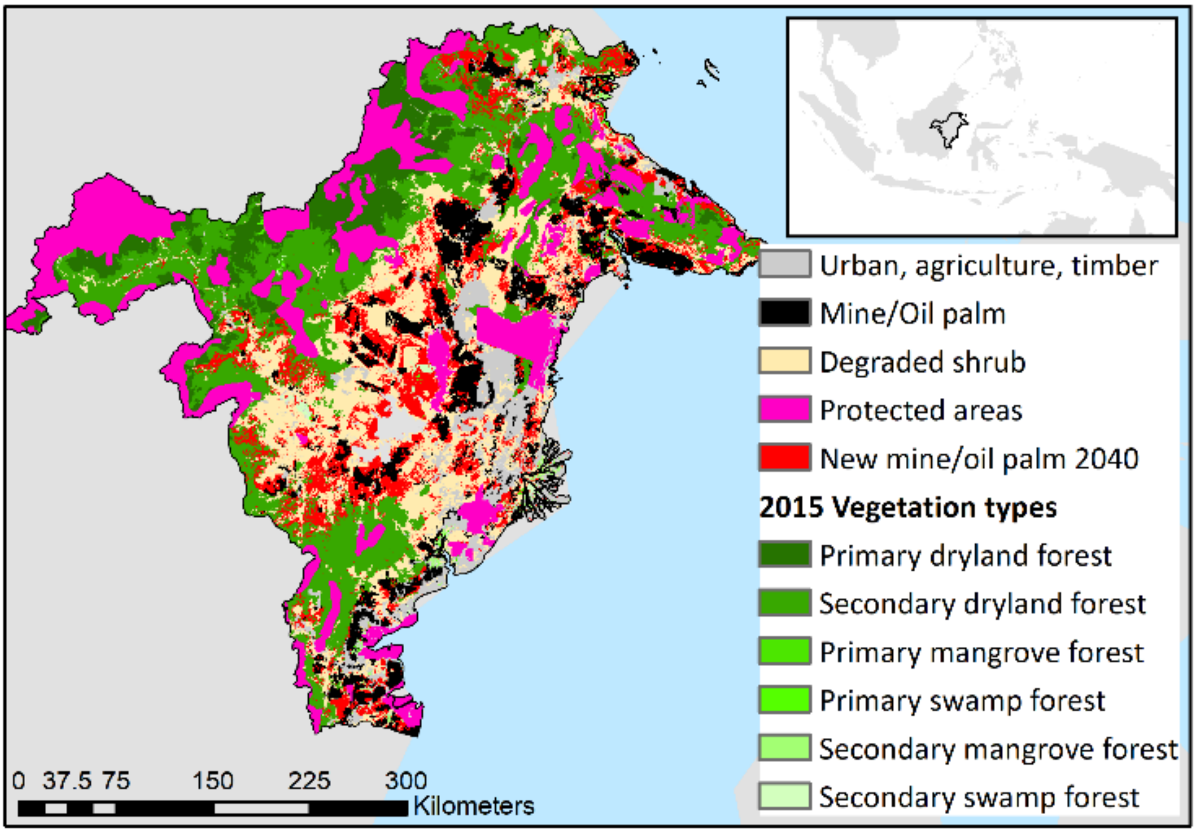

Supplementary Figure 7: East Kalimantan, Indonesia (8.5 Mha). Map shows the extent of future regulated development (new mines and oil palm by 2040) requiring compensation and the current extent of protected areas and native vegetation types (our indicator of biodiversity; see Supplementary Table 11).

Supplementary Table 11: Reclassification of 1996, 2006 and 2015 land use/land cover maps for East Kalimantan. Decisions on how to reclassify data were based on the extent of original classes, the area transitioning between years, and our need to capture regulated and unregulated losses and gains across study region.

**Reclass**	**Original**	**Definition**
Primary forest	Primary dryland forest	Natural tropical forests growing on non-wet substrates, including lowland, upland, and montane forests with no signs of logging activities. The forest includes heath forest and forest on ultramafic and limestone, as well as coniferous, deciduous and mist or cloud forest, which has experienced no (or low) alteration by human activities or logging.
	Primary mangrove forest	Wetland forests in coastal areas such as plains that are influenced by the tides, muddy and brackish water and dominated by species of mangrove and Nipa (*Nipa frutescens*), with no or low influence of human activities or logging.
	Primary swamp forest	Natural tropical forest that grows on wet swamplands, including brackish swamp, marshes, sago, and peat swamp, with no or low influence of human activities or logging.
Secondary forest	Secondary dryland forest	Natural tropical forest growing on non-wet substrates including lowland, upland, and montane forests that exhibit signs of logging activities (indicated by patterns of road development and frequent patches of canopy damage or absence). Secondary forest includes heath forest and forest on ultramafic and limestone, as well as coniferous, deciduous and mist or cloud forest.
	Secondary mangrove forest	Wetland forests in coastal areas such as plains that are influenced by the tides, muddy and brackish water and dominated by species of mangrove and Nipa (*Nipa frutescens*), and exhibit signs of logging activities as described above
	Secondary swamp forest	Natural tropical forest that grows on wet swamp lands, including brackish swamp, marshes, sago, and peat swamp, that exhibit signs of logging activities as described above.
Degraded forest	Dry shrub	Highly degraded areas on non-wet substrates that were previously cleared or heavily logged, and where natural vegetation is regenerating with scattered trees or shrubs.
	Wet shrub	Highly degraded areas on wet substrates (as for swamp or mangrove forests above), that were previously cleared or heavily logged, and where natural vegetation is regenerating with scattered trees or shrubs.
	Bare ground	Areas that are bare of any vegetation, including open exposure areas, craters, sandbanks, sediments, and areas post fire that has not yet exhibit regrowth
Timber	Plantation forest	Forest areas with a regular canopy structure over large areas, dominated by homogeneous tree species. Plantation forests include areas of reforestation, industrial plantation forest and community plantation forest.
Oil palm	Estate crop	Estate areas that have been planted, mostly with perennial crops or other agricultural tree commodities. The majority consist of oil palm plantations.
Urban/rural	Settlement areas	Settlement areas including rural, urban, industrial and other settlements with typical appearance.
	Port and harbour	A port or harbour that is large enough to be delineated as an independent object.
	Transmigration areas	Settlement areas with a unique pattern of association of houses and agroforestry and/or gardens at the peripheries. Part of government programs of migration from other islands to Kalimantan.
Mining	Mining areas	Mining areas exhibit open mining activities such as open-pit mining including tailing grounds and ponds.
Agriculture	Pure dry agriculture	All land covers associated with agricultural activities on dry/non-wet land, such as tegalan (dryland), mixed garden and ladang (agriculture fields)
	Mixed dry agriculture	All land covers associated to agriculture activities on dry/non-wet land that is mixed with shrubs, thickets, and logged over forest. This cover type often results from shifting cultivation and its rotation.
	Paddy field	Rice-farming areas on wet substrates that typically exhibit dyke patterns (pola pematang). This cover type includes rainfed, seasonal paddy field, and irrigated paddy fields.
	Fish pond/aquaculture	Areas exhibit aquaculture activities including fish ponds, shrimp ponds or salt ponds.

Supplementary Table 12: Land use transitions used to calibrate and validate counterfactual model for East Kalimantan.

From	To	Observed		Compensation model
		1996-2006	2006-2015	
Primary	Secondary	792983	91964	Unregulated loss
Secondary	Mining	8253	13442	Regulated loss
	Agriculture	38131	25156	Unregulated loss
	Shrub	981191	250135	Unregulated loss
	Timber	111657	19425	Unregulated loss
	Oilpalm	43599	196304	Regulated loss
Shrub	Secondary	4234	11272	Gains
	Mining	9733	52766	Regulated loss
	Agriculture	30338	100735	Unregulated loss
	Timber	35831	55510	Unregulated loss
	Oilpalm	35454	543902	No loss
Agriculture	Shrub	63268	13158	Gains
	Oilpalm	795	21559	No loss
Timber	Shrub	13683	38144	Gains
Mining	Shrub	1937	17161	Gains

Supplementary Table 13: Spatial determinants used to calibrate land-use change model for East Kalimantan. Variables were not auto correlated.

Variables		Data source
Concessions	Oil palm	[60]
	Mining (gold, coal)	[61]
	Logging	[62]
	Oil and gas	[63]
Biophysical	Elevation	[35]
	Slope	
	Proximity to rivers	[64]
	Orangutan habitat	[65]
	Protected areas	[64]
Socio-economic	Near cleared land	[57]
	Population density	[66]
	Proximity to roads	[67]
	Proximity to villages	[68]

Supplementary Table 14: Model calibration statistics for East Kalimantan. Model performance was calculated as the accuracy of the calibrated model divided by the accuracy of the NULL model.

From	To	Accuracy at 1 ha resolution		
		Calibration	NULL	Performance
Primary	Secondary	0.163	0.037	4.41
Secondary	Mining	0.133	0.003	44.33
	Agriculture	0.050	0.005	10.00
	Shrub	0.092	0.046	2.00
	Timber	0.039	0.004	9.75
	Oil palm	0.189	0.041	4.61
Shrub	Secondary	0.011	0.002	5.50
	Mining	0.153	0.011	13.91
	Agriculture	0.113	0.022	5.14
	Timber	0.080	0.012	6.67
	Oil palm	0.419	0.122	3.43
Agriculture	Shrub	0.030	0.006	5.00
	Oil palm	0.055	0.017	3.24
Timber	Shrub	0.096	0.067	1.43
Mining	Shrub	0.269	0.210	1.28

Supplementary Table 15: Native vegetation extent for historical (1996) and current (2015) landscape conditions East Kalimantan.

Reclass	Vegetation types	Extent (ha)	
		Historical (1996)	Current (2015)
Primary forest	Primary dryland forest	3082160	2182857
	Primary mangrove forest	53852	36528
	Primary swamp forest	46626	22668
Secondary forest	Secondary dryland forest	4925328	4219317
	Secondary mangrove forest	179045	179045
	Secondary swamp forest	157786	115740

Supplementary Table 16: Above ground carbon storage for land use land cover types in East Kalimantan (Supplementary Table 11)[58].

Land use/land cover	Mean (Mg/ha)	Standard deviation
Primary dryland forest	322.10	74.90
Primary mangrove forest	54.21	22.97
Primary swamp forest	211.48	100.83
Secondary dryland forest	257.00	116.48
Secondary mangrove forest	43.22	23.84
Secondary swamp forest	105.11	67.19
Dry shrub	63.42	70.31
Wet shrub	33.44	43.68
Bare ground	38.38	63.04
Mining	14.67	30.05
Oil palm[P]	12.74	23.10

[P] Note: values are slightly lower than those used in Budiharta*, et al.* (39+/-7.4 MgC).

Supplementary Table 17: Crop and practice (C.P) factor used in sediment retention model for East Kalimantan.

Land use/land cover	C.P factor	Source region	Reference
Primary dryland forest	0.001	Malaysia, Philippines	69, 70
Secondary dryland forest	0.033	Malaysia, Philippines, East Kalimantan	69, 70, 71
Primary mangrove forest	0.001	Malaysia	72
Primary swamp forest	0.001	Malaysia	72
Plantation forest	0.13	Indonesia, Philippines	70, 73
Dry shrub	0.0528	Indonesia, Sarawak, Malaysia, Myanmar	72, 73, 74, 75
Estate crop	0.2448	Philippines	69, 70
Settlement areas	0.175	Malaysia, Philippines	70, 72
Bare ground	0.7625	Philippines	70
Secondary mangrove forest	0.033	Malaysia	72
Secondary swamp forest	0.033	Malaysia	72
Wet shrub	0.0119	Malaysia, Tropics	72, 76
Pure dry agriculture	0.2338	Sarawak, Malaysia, Philippines	70, 72, 74
Mixed dry agriculture	0.1441	Malaysia, Philippines, Malaysia	69, 70, 72
Paddy field	0.0695	Malaysia, Philippines, Malaysia	69, 70, 72
Fish pond/aquaculture	0.005	Malaysia	72
Port and harbour	0.175	Malaysia, Philippines	70, 72
Transmigration areas	0.175	Malaysia, Philippines	70, 72
Mining areas	0.9527	Cameron Highlands, Malaysia	72

Supplementary Methods 4: Cabo Delgado, Mozambique

Cabo Delgado is Mozambique's northernmost province, occupying approximately 10% of the country's area (Supplementary Figure 8). It is the second poorest province in Mozambique, with the majority of its approximately 2.3 million inhabitants reliant on subsistence agriculture[77]. While extensive tracts of native vegetation remain, the province is rich in resources such as gemstones, coal and natural gas reserves, and there is substantial attention on developing these industries[78, 79]. Here, we considered a range of potential developments including mining and extractive industries and linear infrastructure (an approximately 220 km road and 300 km power line), the broad footprints of which were obtained from publicly-available sources. We considered these proposed projects to constitute regulated development.

We used 300 m land cover maps (European Space Agency Land Cover Climate Change Initiative (ESA LC CCI) [80] for years 1992, 2004 and 2015 to represent five classes: agriculture, natural vegetation, wetlands and water, settlements, and bare land (Supplementary Table 18). To simulate future land cover change, we incorporated 10 spatial determinants (Supplementary Table 19) relating to two transitions: 1. agriculture to natural vegetation (i.e. regrowth); and 2. natural vegetation to agriculture (unregulated losses) (Supplementary Table 20). We validated the model by simulating transitions between 2004 and 2015 and comparing the simulated map with both the observed 2015 ESA LC CCI map and a null model. The calibrated model performed better than the null model for both transitions (Supplementary Table 21). Using the calibrated model, we simulated land cover change to the year 2040 under the assumption that transition rates were equal to those observed for the period 2004–2015 (Supplementary Table 20) to map counterfactual losses and gains of native vegetation. We used a pre-clearing map of vegetation types[81] to disaggregate current (2015) and future (2040) natural vegetation into vegetation types (Supplementary Table 22).

We obtained data on aboveground carbon storage from a study on ecosystem service trends in Mozambique[82]. We derived current and future maps using average values per land cover class as mapped by the European Space Agency GlobCover product[83] from Niquisse, Cabral[82]. Due to the compatibility of land cover typologies between GlobCover and ESA LC CCI[80], we aligned values from Niquisse, Cabral[82] with land cover classes by ESA LC CCI. One exception was for 'Cropland, irrigated or post-flooding'; data were not presented for this class, so values were derived from Leh, Matlock, Cummings and Nalley (Supplementary Table 23). To create a pre-clearing carbon map, we assigned average carbon values from natural areas to pre-clearing vegetation types (Supplementary Table 22). To calibrate the sediment retention model, we derived crop management (C) and supporting practice (P) factors from Leh, Matlock[84] per land cover class (Supplementary Table 23). The K-factor was derived aligning published values (tonnes per hectare per erosion index unit) for soil classes from east Africa[85] with soil classes in Cabo Delgado[86] (Supplementary Table 25). Additional data included a DEM[87] and rainfall erosivity (based on annual rainfall map) [82, 88].

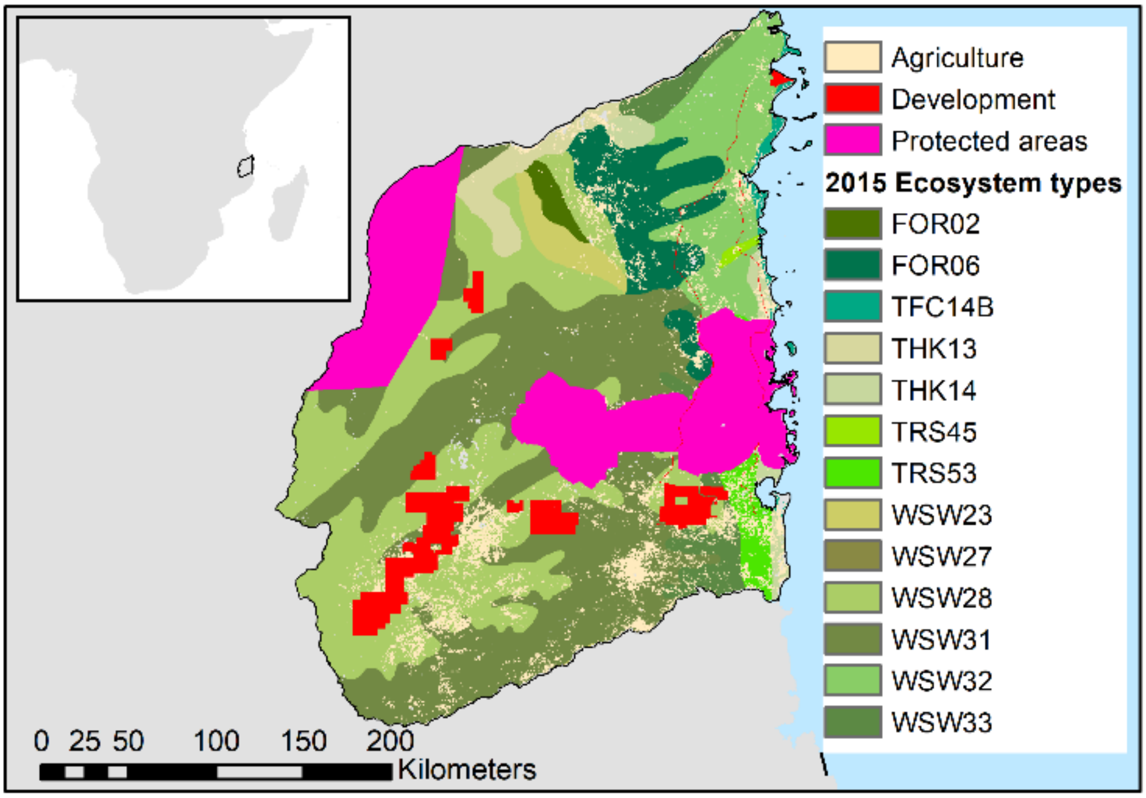

Supplementary Figure 8: Cabo Delgado, Mozambique (7.7 Mha). Map shows the extent of future regulated development (including mining leases and linear infrastructure) requiring compensation and the current extent of protected areas and native vegetation types (our indicator of biodiversity; see Supplementary Table 22). Grey areas within study region boundaries denote no data (generally due to cloud occurrence in land cover maps).

Supplementary Table 18: Reclassification of 1992, 2004 and 2015 ESA LC CCI maps for Cabo Delgado.

**Reclass**	**Original**	**ESA LC CCI map code**
**Agriculture**	Cropland, rainfed	10
	Cropland, rainfed (Herbaceous cover)	11
	Cropland, rainfed (Tree or shrub cover)	12
	Cropland, irrigated or post-flooding	20
	Mosaic cropland (>50%) / natural vegetation (tree, shrub, herbaceous cover) (<50%)	30
	Mosaic natural vegetation (tree, shrub, herbaceous cover) (>50%) / cropland (<50%)	40
**Natural (tree cover, grassland, shrubland)**	Tree cover, broadleaved, evergreen, closed to open (>15%)	50
	Tree cover, broadleaved, deciduous, closed to open (>15%)	60
	Tree cover, broadleaved, deciduous, closed (>40%)	61
	Tree cover, broadleaved, deciduous, open (15-40%)	62
	Tree cover, mixed leaf type (broadleaved and needleleaved)	90
	Mosaic tree and shrub (>50%) / herbaceous cover (<50%)	100
	Mosaic herbaceous cover (>50%) / tree and shrub (<50%)	110
	Shrubland	120
	Deciduous shrubland	122
	Grassland	130
	Sparse vegetation (tree, shrub, herbaceous cover) (<15%)	150
	Tree cover, flooded, fresh or brackish water	160
	Tree cover, flooded, saline water	170
**Wetlands, water**	Shrub or herbaceous cover, flooded, fresh/saline/brackish water	180
	Water bodies	210
**Settlements**	Urban areas	190
**Bare areas**	Bare areas	200
	Unconsolidated bare areas	202

Supplementary Table 19: Spatial determinants used to calibrate land-use change model for Cabo Delgado. Variables were not auto correlated.

Spatial determinants	Data source
Protected areas	[89]
Elevation	[87]
Slope	Calculated using elevation data
Annual average precipitation	[88]
Soil carbon	[90]
Proximity to rivers	[85]
Proximity to roads	[91]
Proximity to towns	[92]
Population density	[93, 94]
Proximity to cleared	Calculated from land cover maps[81] and updated in the model at annual time steps

Supplementary Table 20: Land use transitions used to calibrate and validate counterfactual model for Cabo Delgado. Values in this table are changes in the number of pixels (each pixel is approximately 9.4 ha). All land cover transitions in Cabo Delgado are presented in this table, although only two were considered in the land cover change modelling: Agriculture to Natural; and Natural to Agriculture – these were by far the largest two transitions, and respectively represented regrowth of native vegetation, and unregulated losses of native vegetation.

From	To	Observed		Compensation model
		1992-2004	2006-2015	
Agriculture	Natural	801	2053	Gains
	Settlements	41	101	
Natural (tree cover, grassland and shrubland)	Agriculture	16134	4664	Losses
	Wetlands and water	71	15	
	Settlements	82	175	
	Bare areas	456	109	
Water and wetlands	Agriculture	4	0	
	Natural	126	16	
	Settlements	9	0	
	Bare areas	8	0	
Bare areas	Agriculture	0	1	
	Natural	19	58	
	Settlements	2	8	

Supplementary Table 21: Model calibration statistics for Cabo Delgado. Model performance was calculated as the accuracy of the calibrated model divided by the accuracy of the NULL model.

From	To	Accuracy at 300 m resolution		
		Calibration	NULL	Performance
Agriculture	Natural	0.016	0.010	1.63
Natural	Agriculture	0.029	0.003	8.91

Supplementary Table 22: Native vegetation extent for original and current (2015) landscape conditions in Cabo Delgado. Vegetation types derived from Flora Zambesiaca map[81]. We did not seek to align these vegetation types with specific mapped ESA LC CCI vegetation classes, instead using these vegetation types to represent areas mapped as 'Natural (tree cover, grassland and shrubland)', as per the reclassification of the ESA LC CCI mapping.

Vegetation type	Description	Extent (ha)	
		Historical	Current
FOR02	Moist semi-deciduous forests of the mesoplanaltic slopes and lowlands of the eastern zone (Mozambique)	55529	55106
FOR06	Dry, deciduous, lowland forest	492821	443367
TFC14B	Littoral thicket and forest of recent dunes	90113	68019
THK13	Dry tall mixed thicket (lowland)	177855	161076
THK14	Dry deciduous thicket (sublittoral)	159148	125862
TRS45	Discontinuous dry savanna woodland-tree savanna and "tandos" grassland (Gorongosa lowland)	53262	46745
TRS53	Deciduous tree savanna (lowland, sublittoral)	325528	251978
WSW23	Deciduous miombo savanna woodland	125956	123351
WSW27	Deciduous woodland miombo-discontinuous dry forest-savanna mosaic	470	263
WSW28	Tardily deciduous miombo (north-eastern median altitude) savanna woodland	2113520	1891704
WSW31	Deciduous dry miombo savanna woodland_discontinuous dry savanna (lowland)	3084771	2871072
WSW32	Deciduous miombo savanna woodland - deciduous woodland (north-east sublittoral)	648827	603239
WSW33	Deciduous woodland and thicket_dry deciduous miombo savanna woodland	376684	331293

Supplementary Table 23: Above ground Carbon storage (mg/ha) and crop management (C) and supporting practice (P) factors used in sediment modelling. Data based on ESA descriptions, as cited in Niquisse, Cabral [82] because actual codes differed, unless stated in footnotes.

Code	Description	Carbon	C.P factor
10[a]	Cropland, rainfed	9.3	0.500
11[a]	Cropland, rainfed (Herbaceous cover)	9.3	0.500
12[a]	Cropland, rainfed (Tree or shrub cover)	9.3	0.500
20[b]	Cropland, irrigated or post-flooding	7.4	0.200
30	Mosaic cropland (>50%) / natural vegetation (tree, shrub, herbaceous cover) (<50%)	116.6	0.250
40	Mosaic natural vegetation (tree, shrub, herbaceous cover) (>50%) / cropland (<50%)	61.3	0.300
50[c]	Tree cover, broadleaved, evergreen, closed to open (>15%)	181.6	0.005
60[c]	Tree cover, broadleaved, deciduous, closed to open (>15%)	181.6	0.005
61	Tree cover, broadleaved, deciduous, closed (>40%)	227.0	0.001
62	Tree cover, broadleaved, deciduous, open (15-40%)	147.6	0.010
90	Tree cover, mixed leaf type (broadleaved and needleleaved)	158.9	0.005
100	Mosaic tree and shrub (>50%) / herbaceous cover (<50%)	88.7	0.025
110	Mosaic herbaceous cover (>50%) / tree and shrub (<50%)	82.0	0.059
120[d]	Shrubland	30.0	0.080
122[d]	Deciduous shrubland	30.0	0.080
130	Grassland	50.8	0.080
150	Sparse vegetation (tree, shrub, herbaceous cover) (<15%)	40.7	0.200
160	Tree cover, flooded, fresh or brakish water	136.2	0.014
170	Tree cover, flooded, saline water	115.8	0.050
180	Shrub or herbaceous cover, flooded, fresh/saline/brakish water	50.8	0.077
190	Urban areas	3.0	0.100
200	Bare areas	2.3	0.350
202	Unconsolidated bare areas	2.3	0.350
210	Water bodies	0	0.010

[a] Niquisse et al. 2017 provided an average value for rainfed cropland, which we applied to classes 10–12.
[b] Niquisse et al. 2017 coded irrigated cropland as class 11; we applied this value to class 20.
[c] Niquisse et al. 2017 averaged value broadleaved deciduous forests, which we applied to classes 50 and 60.
[d] Niquisse et al. 2017 provided an average value for all shrubland, which we applied to classes 120 and 122.

Supplementary Table 24: Average above ground carbon storage (Mg/ha) within natural areas (category 2) by vegetation type (see Supplementary Table 23).

**Floralegen**	**Carbon**
FOR02	172.63
FOR06	159.48
TFC14B	98.45
THK13	141.61
THK14	135.23
TRS45	135.57
TRS53	134.24
WSW23	165.58
WSW27	69.21
WSW28	99.94
WSW31	85.22
WSW32	150.47
WSW33	143.43

Supplementary Table 25: Soil erodibility values for Cabo Delgado, derived by linking erodibility values from Kenya[85] to Harmonized World Soil Database mapping units[86].

**Soil unit**	**Soil texture class**	**K-factor (t/ha per erosion index unit)**
Haplic Acrisols	Sandy clay loam	0.18
Luvic Arenosols	Sand	0.18
Luvic Arenosols	Sand	0.18
Cambic Arenosols	Sand	0.18
Albic Arenosols	Sand	0.18
Eutric Cambisols	Sandy loam	0.28
Eutric Cambisols	Sandy clay loam	0.18
Ferralic Cambisols	Sandy clay	0.11
Salic Fluviosls	Sand	0.28
Mollic Fluvisols	Clay (light)	0.18
Mollic Gleysols	Sandy clay loam	0.11
Eutric Leptosols	Sandy loam	0.28
Eutric Leptosols	Loam	0.42
Eutric Leptosols	Loam	0.42
Haplic Lixisols	Loamy sand	0.28
Haplic Lixisols	Sandy loam	0.28
Haplic Lixisols	Sandy clay loam	0.18
Haplic Lixisols	Sandy clay loam	0.18
Haplic Lixisols	Sandy loam	0.28
Haplic Lixisols	Sandy loam	0.28
Haplic Lixisols	Sandy loam	0.28
Haplic Luvisols	Sandy loam	0.18
Haplic Luvisols	Sandy loam	0.18
Chromic Luvisols	Sandy loam	0.18
Luvic Phaeozems	Clay (light)	0.11
Eutric Vertisols	Clay (heavy)	0.42

REFERENCES

1. Seabrook L, McAlpine C, Fensham R. Cattle, crops and clearing: Regional drivers of landscape change in the Brigalow Belt, Queensland, Australia, 1840–2004. *Landsc. Urban Plan.* **78**, 373-385 (2006).

2. Bowen M. Quantifying the ecological values of brigalow regrowth for woodland birds: a hierarchical landscape approach (2009).

3. Bowen ME, McAlpine CA, House APN, Smith GC. Regrowth forests on abandoned agricultural land: A review of their habitat values for recovering forest fauna. *Biol. Conserv.* **140**, 273-296 (2007).

4. Queensland Government. *Vegetation Management Act* (1999).

5. DSEWPC. *EPBC Act List of Threatened Ecological Communities*. Australian Government, Department of Sustainability, Environmental, Water, Population and Communities (DSEWPC) (2008).

6. DDIP. *State Development Areas - Queensland*. Queensland Government, Department of Development, Infrastructure and Planning (DDIP) (2014).

7. Commonwealth of Australia. *EPBC Act Environmental Offsets Policy*. Australian Government, Department of Sustainability, Environmental, Water, Population and Communities (2012).

8. Queensland Government. *Environmental Offsets Policy* (2014).

9. DSITI. *Land cover change in Queensland: a Statewide Landcover and Trees Study (SLATS) report*. Queensland Department of Science, Information Technology and Innovation (2015).

10. Sonter LJ, Tomsett N, Wu D, Maron M. Biodiversity offsetting in dynamic landscapes: Influence of regulatory context and counterfactual assumptions on achievement of no net loss. *Biol. Conserv.* **206**, 314-319 (2017).

11. Simmons BA, Law EA, Marcos-Martinez R, Bryan BA, McAlpine C, Wilson KA. Spatial and temporal patterns of land clearing during policy change. *Land Use Policy* **75**, 399-410 (2018).

12. Queensland Herbarium. *Regional Ecosystem Description Database (REDD). Version 9.0*. Queensland Government, Department of Science, Information Technology and Innovation (DSITI) (2015).

13. Barrett DJ. Steady state turnover time of carbon in the Australian terrestrial biosphere. *Global Biogeochem. Cycles* **16**, 55-51-55-21 (2002).

14. Barrett DJ. Timescales and Dynamics of Carbon in Australia's Savannas. In: *Ecosystem Function in Savannas : Measurement and Modeling at Landscape to Global Scales* (eds Hanan NP, Hill MJ). Boca Raton, US: CRC Press (2010).

15. Gallant JC, Dowling TI, Read AM, Wilson N, Tickle P, Inskeep C. 1 second SRTM Derived Digital Elevation Models User Guide. Geoscience Australia (2011).

16. DSITI. Soils - universal soil loss equation series. Department of Science ITaI, State of Queensland. Queensland Spatial Catalogue (2016).

17. DSITI. Land use mapping - 1999 to Current - Queensland. Department of Science ITaI, State of Queensland (2017).

18. DNRM. Drainage basin sub-area Queensland. Department of Natural Resources and Mines SoQ (2009).

19. Teng H, Viscarra Rossel R, Shi Z, Behrens T, Chappell A, Bui E. Assimilating satellite imagery and visible-near infrared spectroscopy to model and map soil loss by water erosion in Australia. *Environ. Model. & Softw.* **77**, 156-167 (2016).

20. Threatened Species Scientific Commitee. Brigalow (Acacia harpophylla dominant and co-dominant) recommendation to the Minister for the Environment and Water Resources. In: *from the Threatened Species Scientific Committee (TSSC) on a public nomination for an ecological community listing on the Environment Protection and Biodiversity Conservation Act 1999* (2001).

21. Isbell RF, National Committee on Soil and Terrain. *The Australian Soil Classification*. CSIRO Publishing (2014).

22. Geoscience Australia. *Digital Elevation Model (DEM-H) 3 second - Queensland*. Commonwealth of Australia (2014).

23. Pitney B. *Streetpro Australia version 10*. Pitney Bowes Software Australia Pty Ltd. (2009).

24. InfoMine. Company & Property Mine. InfoMine (2012).

25. Ribeiro MC, Metzger JP, Martensen AC, Ponzoni FJ, Hirota MM. The Brazilian Atlantic Forest: How much is left, and how is the remaining forest distributed? Implications for conservation. *Biol. Conserv.* **142**, 1141-1153 (2009).

26. Sonter LJ, Barrett DJ, Soares-Filho BS, Moran CJ. Global demand for steel drives extensive land-use change in Brazil's Iron Quadrangle. *Glob. Environ. Change* **26**, 63-72 (2014).

27. Jacobi CM, do Carmo FF. The contribution of ironstone outcrops to plant diversity in the Iron Quadrangle, a threatened Brazilian landscape. *Ambio* **37**, 324-326 (2008).

28. Silveira FAO*, et al.* Ecology and evolution of plant diversity in the endangered campo rupestre: a neglected conservation priority. *Plant and Soil* **403**, 129-152 (2016).

29. Brasil. Lei nº 11.428: A utilização e proteção da vegetação nativa do Bioma Mata Atlântica, e dá outras providências. (2006).

30. Brasil. Disp˜oe sobre a protec¸˜ao da vegetac¸˜ao nativa; altera as Leis nos 6938, 9393, e 11428; revoga as Leis nos 4771, e 7754, e a Medida Provis´oria no 2166–67; e d´a outras provid ˆencias. Casa Civil SpAJı (2012).

31. IBGE. Base Cartográfica Contínua, ao milionésimo – BCIM: versão 3. Instituto Brasileiro de Geografia e Estatística (IBGE) (2010).

32. MME. Plano Nacional de Mineração 2030: Geologia, Mineração e Transformação Mineral. Ministério de Minas e Energia (2011).

33. IBGE. Base Cartográfica Contínua, ao milionésimo – BCIM: Historico. Instituto Brasileiro de Geografia e Estatística (IBGE) (2003).

34. Soares-Filho BS*, et al.* Brazil's Market for trading forest certificates. *PLOS ONE* **11**, e0152311, https://doi.org/10.1371/journal.pone.0152311 (2016).

35. USGS. Shuttle Radar Topography Mission (SRTM) Global 1 arc second V003. (Global Land Cover Facility UoM) (2006).

36. Moreira MC, Pruski FF, Oliveira TECd, Pinto FdAdC, Silva DDd. NetErosividade MG: erosividade da chuva em Minas Gerais. *Revista Brasileira de Ciência do Solo* **32**, 1349-1353 (2008).

37. Duarte GT, Ribeiro MC, Paglia AP. Ecosystem Services Modeling as a Tool for Defining Priority Areas for Conservation. *PLOS ONE* **11**, e0154573, https://doi.org/10.1371/journal.pone.0154573 (2016).

38. Embrapa. Mapa de solos do estado de Minas Gerais. Embrapa (2004).

39. Veloso HP, Rangel Filho ALR, Lima JCA. Classificação daVegetação Brasileira, adaptada a um sistema universal. MEFP/IBGE/DRNEA,RJ. (1991).

40. IBAMA. Unidades de Conservação. Instituto Brasileiro do Meio Ambiente e dos Recursos Naturais Renováveis (IBAMA) (2010).

41. DNPM. Processos Minerarios: Sistema de Informações Geográficas da Mineração (SIGMINE) Departamento Nacional de Produção Mineral (DNPM) (2017).

42. Beskow S, Mello CR, Norton LD, Curi N, Viola MR, Avanzi JC. Soil erosion prediction in the Grande River Basin , Brazil using distributed modeling. *Catena* **79**, 49–59 (2009).

43. Bloise GDLF, Júnior OAdC, Reatto A, Guimarães RF, Martins EdS, Carvalho APF. Evaluation of natural susceptibility to erosion of the Olaria basin. *Planaltina*, 33 (2001).

44. Correchel V, Bacchi OOS, De Maria IC, Dechen SCF, Reichardt K. Erosion rates evaluated by the 137Cs technique and direct measurements on long-term runoff plots under tropical conditions. *Soil Tillage Res.* **86**, 199–208 (2006).

45. Mannigel AR, Carvalho MdP, Moreti D, Luciano dRM. Erodibility factor and loss tolerance of soils of São Paulo state. *Acta Sci* **24**, 1335–1340 (2002).

46. Silva A.M. da, Silva M.L.N., Curi N., Avanzi J.C., Ferreira M.M. Rainfall erosivity and erodibility of Cambisol and Latosol in the region of Lavras, south of Minas Gerais. *Rev Bras Ciência do Solo* **33**, 1811–1820 (2009).

47. Silva MLN, Curi N, Oliveira MS, Ferreira MM, Neto FL. Comparison between direct and indirect methods for determination of erodibility in oxisols under Cerrado. *Pesqui agropecuária Bras* **29**, 1751–1761 (1994).

48. IBGE. Mapa de Solos do Brasil. Instituto Brasileiro de Geografia e Estatística (IBGE) (2006).

49. D'Andréa AF, Silva MLN, Curi N, Guilherme LRG. Carbon and nitrogen stock and forms of mineral nitrogen in soil under different management systems. *Pesqui agropecuária Bras* **39**, 179–186 (2004).

50. Silva MA. Modeling spatial of water erosion in the Doce River Valley, Central-East region of Minas Gerais state – Brazil. Universidade Federal de Lavras (2009).

51. Yang D, Kanae S, Oki T, Koike T, Musiake K. Global potential soil erosion with reference to land use and climate changes. *Hydrol Process* **17**, 2913–2928 (2003).

52. Alamgir M, Campbell MJ, Sloan S, Suhardiman A, Supriatna J, Laurance WF. High-risk infrastructure projects pose imminent threats to forests in Indonesian Borneo. *Sci. Rep.* **9**, 140 (2019).

53. Margono BA, Potapov PV, Turubanova S, Stolle F, Hansen MC. Primary forest cover loss in Indonesia over 2000-2012. *Nat. Clim. Change* **4**, 730-735 (2014).

54. Gaveau DLA*, et al.* Rapid conversions and avoided deforestation: examining four decades of industrial plantation expansion in Borneo. *Sci. Rep.* **6**, 32017 (2016).

55. Budiharta S, *et al.* Restoration to offset the impacts of developments at a landscape scale reveals opportunities, challenges and tough choices. *Glob. Environ. Change* **52**, 152-161 (2018).

56. Indonesia PMPR. Sistem Sertifikasi Kelapa Sawit Berkelanjutan Indonesia (Indonesian Sustainable Palm Oil Certification System/ISPO). Kementerian Pertanian Republik Indonesia (2015).

57. MoEF. Indonesia's Land Cover Map. Forestry Planning Agency IMoF (2016). Available at: http://webgis.menlhk.go.id:8080/kemenhut/index.php/id/peta/peta-interaktif

58. Ferraz A, *et al.* Carbon storage potential in degraded forests of Kalimantan, Indonesia. *Environ. Res. Lett.* **13**, 095001 (2018).

59. Panagos P, *et al.* Global rainfall erosivity assessment based on high-temporal resolution rainfall records. *Sci. Rep.* **7**, 4175 (2017).

60. East Kalimantan Estate Crops Office. Oil Palm Concessions. (2016). Available at: https://geospasial-perkebunan.kaltimprov.go.id/sigbun01/index.php/home

61. East Kalimantan Mining and Energy Office. Mining Concessions. (2017). Available at: https://momi.minerba.esdm.go.id/public/

62. East Kalimantan Forestry Office. Logging Concessions. (2017). Available at: http://webgis.menlhk.go.id:8080/kemenhut/index.php/id/peta/peta-interaktif

63. Ministry of Energy and Mining. Oil and Gas Concessions. (2017). Available at: https://momi.minerba.esdm.go.id/public/

64. Government of East Kalimantan. Provincial Spatial Plan 2016-2036 (RTRWP: Rencana Tata Ruang Wilayah Propinsi). (2012). Available at: https://geospasial-perkebunan.kaltimprov.go.id/sigbun01/index.php/home

65. Santika T, *et al.* First integrative trend analysis for a great ape species in Borneo. *Sci. Rep.* **7**, 4839 (2017).

66. WorldPop. Number of people per pixel. (2015).

67. BIG: Badan Informasi Geospasial (National Geospatial Information Agency). Topographic Maps. (2017).

68. East Kalimantan Planning Office Villages. (2016).

69. Morgan RPC. Soil Erosion and Conservation. Longman Group Limited (1995).

70. David WF. Soil and Water Conservation Planning: Policy Issues and Recommendations. *Journal of Philippine Development,* **15**, 47-84 (1988).

71. Ellis P, Griscom B, Walker W, Gonçalves F, Cormier T. Mapping selective logging impacts in Borneo with GPS and airborne lidar. *For. Ecol. and Manag.* **365**, 184–196 (2016).

72. Teh SH. Soil Erosion Modeling using RUSLE and GIS on Cameron Highlands, Malaysia for Hydropower Development. University of Iceland & University of Akureyri (2011).

73. Asdak C. *Hidrologi dan Pengelolaan Daerah Aliran Sungai*. Gadjah Mada University Press (1995).

74. Vijith H, Seling LW, Dodge-Wan D. *Estimation of soil loss and identification of erosion risk zones in a forested region in Sarawak, Malaysia, Northern Borneo*. Springer Netherlands. (2017).

75. Wolny S, Hamel P, Mandle L. Myanmar National Ecosystem Service Assessment Technical Report. Stanford, CA: Natural Capital Project (2016).

76. Qin Y, Gartner T, Minnemeyer S, Reig P, Sargent S. Global Forest Watch Water Metadata Document. (2016).

77. Lalani B, Dorward P, Holloway G. Farm-level Economic Analysis - Is Conservation Agriculture Helping the Poor? *Ecol. Econ.* **141**, 144-153 (2017).

78. Symons K. Transnational spaces, hybrid governance and civil society contestation in Mozambique's gas boom. *Extr. Ind. Soc.* **3**, 149-159 (2016).

79. Wiegink N. Imagining Booms and Busts: Conflicting Temporalities and the Extraction-"Development" nexus in Mozambique. *Extr. Ind. Soc.* **5**, 245-252 (2018).

80. European Space Agency. Land Cover CCI Product User Guide Version 2.0. (2017).

81. Wild H, Grandvaux Barbosa LA. Vegetation Map (1:2,500,000 in Colour) of the Flora Zambesiaca Area. M.O. Collins Pty Ltd (1967).

82. Niquisse S, Cabral P, Rodrigues Â, Augusto G. Ecosystem services and biodiversity trends in Mozambique as a consequence of land cover change AU - Niquisse, Sérgio. *Int. J. Biodivers. Sci. Ecosyst. Serv. Manag*. **13**, 297-311 (2017).

83. European Space Agency. GlobCover. (2009).

84. Leh MDK, Matlock MD, Cummings EC, Nalley LL. Quantifying and mapping multiple ecosystem services change in West Africa. *Agric. Ecosyst. Environ.* **165**, 6-18 (2013).

85. Kassam AH, van Velthuizen, H.T., Mitchell, AJ.B., Fischer, G.W., and Shah, M.M., . Agro-ecological land resources assessment for agricultural development planning – a case study of Kenya – Technical Annex 2. Food and Agriculture Organization of the United Nations and International Institute for Applied Systems Analysis (1992).

86. FAO. Harmonized World Soil Database (version 1.2). 2009.

87. National Aeronautic and Space Administration, Ministry of Economy T, and Industry,. ASTER Global Digital Elevation Model (GDEM) v2 data. (2011).

88. Fick SE, Hijmans RJ. Worldclim 2: New 1-km spatial resolution climate surfaces for global land areas. *Int. J. Climatol.* **37**, 4302–4315 (2017).

89. UNEP-WCMC, IUCN. Protected Planet: The World Database on Protected Areas (WDPA). (2018).

90. Hengl T*, et al.* SoilGrids250m: Global gridded soil information based on machine learning. *PLOS ONE* **12**, e0169748 (2017).

91. Center for International Earth Science Information Network - CIESIN - Columbia University and Information Technology Outreach Services - ITOS - University of Georgia. Global Roads Open Access Data Set, Version 1 (gROADSv1). (2013).

92. DINAGECA. Cabo Delgado villages in 2006. National Directorate of Geography and Cadastre (2006).

93. Linard C, Gilbert M, Snow RW, Noor AM, Tatem AJ. Population Distribution, Settlement Patterns and Accessibility across Africa in 2010. *PLOS ONE* **7**, e31743 (2012).

94. WorldPop. Mozambique 100 m population. (2013).
