# Supplementary material for: Local conditions and policy design determine whether ecological compensation can achieve No Net Loss goals
Source: Nat Commun. 2020 Apr 29;11:2072. doi: 10.1038/s41467-020-15861-1 (PMC7190705; doi:10.1038/s41467-020-15861-1)
Supplement: Supplementary file 3 — Description of Additional Supplementary Information [file 41467_2020_15861_MOESM3_ESM.pdf]

Description of Additional Supplementary Files

File Name: Supplementary Dataset 1

Description: Impacts on biodiversity and ecosystem services. Results show net impacts of regulated development and 18 compensation policy design options on vegetation types (our indicator of biodiversity) and two ecosystem services (carbon storage and sediment retention). Impacts from development are net losses (i.e. negative values) and impact from compensation are net gains (i.e. positive values). Thus compensation achieves No Net Loss when compensation impacts are greater than those from regulated development. Results for each case study (Brigalow Belt, Iron Quadrange, East Kalimantan, Cabo Delgado) is shown on a separate tab.
